# Variation by Institution in Sexual Harassment Experiences Among US Medical Interns

**DOI:** 10.1001/jamanetworkopen.2023.49129

**Published:** 2023-12-26

**Authors:** Elizabeth M. Viglianti, Andrea L. Oliverio, Karina Pereira-Lima, Elena Frank, Lisa M. Meeks, Srijan Sen, Amy S. B. Bohnert

**Affiliations:** 1 Division of Pulmonary and Critical Care Medicine, University of Michigan, Ann Arbor; 2 Division of Nephrology, University of Michigan, Ann Arbor; 3 Department of Neurology, University of Michigan, Ann Arbor; 4 Michigan Neuroscience Institute, University of Michigan, Ann Arbor; 5 Department of Learning Health Sciences and Family Medicine, University of Michigan, Ann Arbor; 6 Eisenberg Family Depression Center, University of Michigan, Ann Arbor; 7 Department of Anesthesiology, University of Michigan, Ann Arbor

## Abstract

This cross-sectional study investigates possible institutional and specialty variations in experiences of sexual harassment among US medical interns.

## Introduction

Sexual harassment is experienced by 20% to 25% of US women resident physicians.^1,2^ Most studies of sexual harassment are single center or single specialty, limiting the understanding of variation across institutions and specialties.^3,4^ The purpose of this cross-sectional study was to investigate possible institutional variation in experiences of sexual harassment among US medical interns.

## Methods

The cross-sectional study followed the STROBE reporting guideline and was approved by the University of Michigan Institutional Review Board. Participants provided electronic informed consent.

We analyzed data from June 2016 to June 2017 in the Intern Health Study, an ongoing National Institutes of Health–funded repeated cohort study of postgraduate year 1 residents (interns) and the Association of American Medical Colleges (AAMC). Participants completed the shortened Sexual Experiences Questionnaire (SEQ-S)^5^ at the end of their intern year (eMethods 1 in Supplement 1). Sexual harassment was defined as endorsing at least 1 sexual harassment item of the SEQ-S. Surveys were linked with AAMC data to obtain institution characteristics. Institutions with fewer than 50 respondents and respondents who identified as nonbinary were excluded from the primary analysis due to small sample size.

Self-reported demographic characteristics are presented as counts (percentages) or medians (IQR). We used a 2-level hierarchical logistic regression model to assess institutional variation in intern experiences of any form of sexual harassment (primary outcome) across institutions, adjusting for age, self-reported race and ethnicity (associated with increased rates of discrimination and sexual harassment), and sex. We quantified the variation in the probability of self-reporting sexual harassment across institutions using the intraclass correlation coefficient (ICC) and the median odds ratio (MOR), which were calculated from the final model using the method of Merlo.^6^ A sensitivity analysis was performed using similar methods to assess variation in sexual harassment among specialty training programs (eMethods 2 in Supplement 1). Analyses were performed from March 13 to October 23, 2023, using Stata, version 15.1 (StataCorp LLC) with significance set at 2-sided *P* < .05.

## Results

Of the 6134 interns from 360 institutions who initiated the survey, 2027 from 28 institutions were included in the analysis (963 men [47.5%] and 1064 women [52.5%]; median age, 27 [IQR, 26-28]); they were predominantly Asian (403 [19.9%]), White (1247 [61.5%]), or multiracial (178 [8.8%]) (Table). Sexual harassment was experienced by 1311 interns (64.7%), including 821 women (77.2%) and 490 men (50.9%).

**Table.** Demographic Characteristics of Interns by Self-Reported Experiences of Sexual Harassment

Characteristic	Participant group^a^	
	Experienced sexual harassment (n = 1311)	Did not experience sexual harassment (n = 746)
Age, median (IQR)	27 (26-28)	27 (26-28)
Sex		
Women	821 (77.2)	243 (22.8)
Men	490 (50.9)	473 (49.1)
Race and ethnicity		
Arab or Middle Eastern	18 (58.1)	13 (41.9)
Asian	252 (62.5)	151 (37.5)
URIM^b^	92 (62.2)	56 (37.8)
White	813 (65.2)	434 (34.8)
Multiracial	121 (68.0)	57 (32.0)
Other or unknown^c^	15 (75.0)	5 (25.0)

Abbreviation: URIM, underrepresented in medicine.

^a^ Unless otherwise indicated, data are expressed as No. (row %) of participants.

^b^ Includes interns who self-identified as American Indian or Alaska Native, Black or African American, Hispanic, or Native Hawaiian or Other Pacific Islander.

^c^ Other was self-reported by the intern; unknown indicates no race or ethnicity was reported by the intern.

After adjusting for respondent characteristics, there was significant variation between institutions in the prevalence of sexual harassment (ICC, 0.01 [95% CI, 0.003-0.05]) with an absolute difference of 12.2% between institutions for low and high prevalence. The MOR was 1.20 (95% CI, 1.09-1.45), meaning for 2 interns with the same characteristics, the intern at the institution with a higher prevalence of sexual harassment would have 20% greater odds of experiencing sexual harassment (Figure). There was also significant variation in sexual harassment among the 9 training specialties in the fully adjusted model (ICC, 0.01 [95% CI, 0.003-0.05]) with an MOR of 1.22 (95% CI, 1.10-1.52) (Figure).

**Figure.**
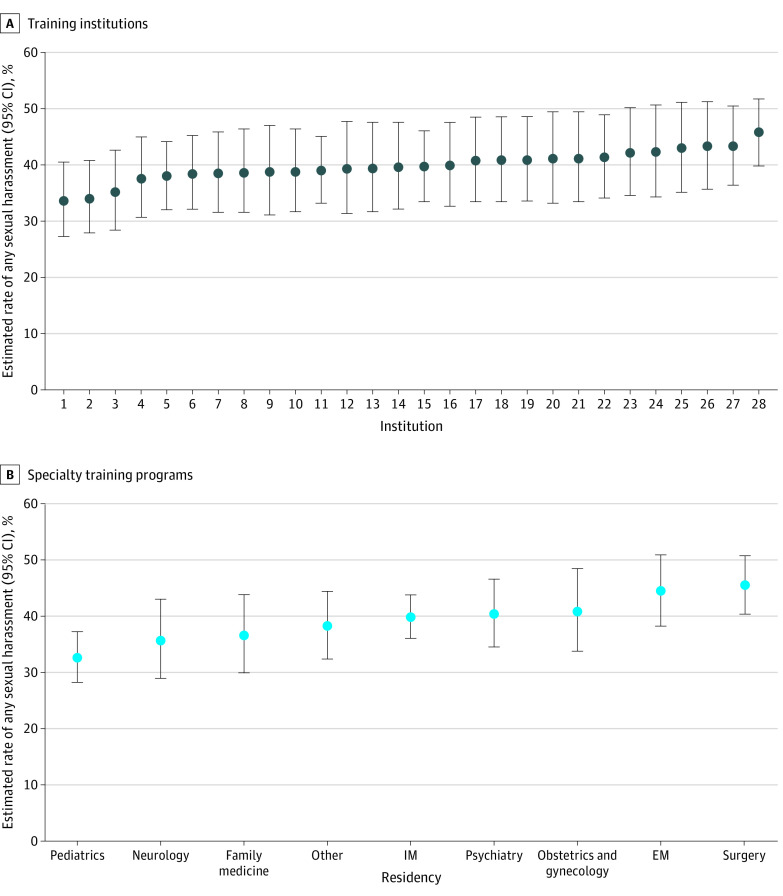
Caterpillar Plots of Variation in Experiences of Sexual Harassment Across Training Institutions and Specialty Programs Specialties categorized as other included transitional, pathology, dermatology, and physical medicine and rehabilitation. EM indicates emergency medicine; IM, internal medicine.

## Discussion

Among a US national cohort of interns, over half experienced sexual harassment. Although harassment was prevalent across programs, institutional and specialty training variations in interns’ sexual harassment experiences exist, thereby providing additional evidence that residency programs and institutions play an important role in combating this widespread problem.

This study builds on prior work limited to surgical trainees noting variation in program-level rates of sexual harassment.^1,4^ Limitations include the binary definition of sex, lack of generalizability outside of the US, and potential responder bias. Further work is necessary to understand cultural or policy differences that influence the rates of sexual harassment within institutions and specialties. This data could inform interventions and facilitate the sharing of best practices, ultimately reducing the unacceptably high frequency of sexual harassment experienced by resident physicians.

## Notes

Supplement 1. **eMethods 1.** Intern Health Study and Survey Questions **eMethods 2.** Detailed Statistical Approach
eReference

Supplement 2.
Data Sharing Statement

## References

[1] Hu YY, Ellis RJ, Hewitt DB, . Discrimination, abuse, harassment, and burnout in surgical residency training. N Engl J Med. 2019;381(18):1741-1752. doi:10.1056/NEJMsa1903759 31657887 PMC6907686

[2] Viglianti EM, Meeks LM, Oliverio AL, Lee KT, Iwashyna TJ, Hingle TJ. Self-reported sexual harassment and subsequent reporting among internal medicine residency trainees in the United States. JAMA Intern Med. 2023;183(3):269-271. doi:10.1001/jamainternmed.2022.6108 36648861 PMC9857703

[3] Viglianti EM, Oliverio AL, Cascino TM, Meeks LM. The Policy gap: a survey of patient-perpetrated sexual harassment policies for residents and fellows in prominent US hospitals. J Gen Intern Med. 2019;34(11):2326-2328. doi:10.1007/s11606-019-05229-7 31414357 PMC6848723

[4] Schlick CJR, Ellis RJ, Etkin CD, . Experiences of gender discrimination and sexual harassment among residents in general surgery programs across the US. JAMA Surg. 2021;156(10):942-952. doi:10.1001/jamasurg.2021.3195 34319377 PMC8319819

[5] Vargas EA, Brassel ST, Cortina LM, Settles IH, Johnson TRB, Jagsi R. #MedToo: A Large-Scale Examination of the Incidence and Impact of Sexual Harassment of Physicians and Other Faculty at an Academic Medical Center. J Womens Health (Larchmt). 2020;29(1):13-20. doi:10.1089/jwh.2019.7766 31513467

[6] Merlo J, Chaix B, Ohlsson H, . A brief conceptual tutorial of multilevel analysis in social epidemiology: using measures of clustering in multilevel logistic regression to investigate contextual phenomena. J Epidemiol Community Health. 2006;60(4):290-297. doi:10.1136/jech.2004.029454 16537344 PMC2566165

