# Supplementary material for: Variation by Institution in Sexual Harassment Experiences Among US Medical Interns
Source: JAMA Netw Open. 2023 Dec 26;6(12):e2349129. doi: 10.1001/jamanetworkopen.2023.49129 (PMC10751587; doi:10.1001/jamanetworkopen.2023.49129)
Supplement: Supplement 1. — eMethods 1. Intern Health Study and Survey Questions eMethods 2. Detailed Statistical Approach eReference [file jamanetwopen-e2349129-s001.pdf]

Supplementary Online Content

Viglianti EM, Oliverio AL, Pereira-Lima K, et al. Variation by institution in sexual harassment experiences among US interns. *JAMA Netw Open*. 2023;6(12):e2349129. doi:10.1001/jamanetworkopen.2023.49129

eMethods 1. Intern Health Study and Survey Questions
eMethods 2. Detailed Statistical Approach
eReference

This supplementary material has been provided by the authors to give readers additional information about their work.

© 2023 Viglianti EM et al. *JAMA Network Open*.

eMethods 1. Intern Health Study and Survey Questions

Recruitment strategy

The present study was performed as part of the Intern Health Study,[1] an NIH-funded longitudinal cohort study that assesses stress and mood in medical interns at institutions around the US and has been conducted annually since 2007. Email addresses for incoming first-year residents across all specialties throughout the United States were gathered from residency programs and publicly available databases. Eligible residents were invited via email to complete a baseline survey two months prior to internship start and quarterly surveys during their internship year. The sexual harassment questions were included in the fourth quarterly survey for the 2016 and 2017 cohorts.

Survey questions

Baseline questions used in the present study

- Date of Birth (MM/DD/YYYY) _____________
- Sex assigned at birth
  - Male
  - Female
- Ethnicity (check all that apply)
  - Arab/Middle Eastern
  - Asian
  - Black/African American
  - Latino/Hispanic
  - Native American
  - Pacific Islander
  - White
  - Other ___________

© 2023 Viglianti EM et al. *JAMA Network Open*.

- Residency specialty
  - Internal Medicine
  - Surgery
  - Obstetrics/Gynecology
  - Pediatrics
  - Psychiatry
  - Emergency Medicine
  - Med/Peds
  - Family Medicine
  - Anesthesiology (without transitional year)
  - Neurology (without transitional year)
  - Otolaryngology (without transitional year)
  - Transitional year
  - Other ______________________

End-of-year (12-month of internship) questions

Sexual Experiences Questionnaire Shortened (SEQ-S)

1. Since graduating medical school and during your post-graduate training, were you ever sexually harassed?
   a. Yes
   b. No

   If yes, how were you harassed?

The following questions had the optional responses of: Never, Once, Sometimes, Often, Very often

Since starting your residency, have you ever been in a situation where any individuals…

2. Habitually told suggestive stories or offensive jokes?

3. Made unwanted attempts to draw you into a discussion of personal or sexual matters (e.g., attempted to discuss or comment on your sex life)?

© 2023 Viglianti EM et al. *JAMA Network Open*.

4. Made crude and offensive sexual remarks, either publicly (e.g., in the office), or to you privately?
5. Treated you “differently” because of your sex (e.g., mistreated, slighted, or ignored you)?
6. Gave you unwanted sexual attention?
7. Displayed, used, or distributed sexist or suggestive materials (e.g., pictures, stories, or pornography)?
8. Frequently made sexist remarks (e.g., suggesting that women are too emotional to be scientists or that men should not be the primary caretakers of children because they are not nurturing)?
9. Attempted to establish a romantic relationship with you despite your efforts to discourage this person?
10. “Put you down” or was condescending to you because of your sex?
11. Has continued to ask you for a date, drinks, dinner, etc., even though you have said “no”?
12. Made you feel like you were being subtly bribed with some sort of reward or special treatment to engage in sexual behavior?
13. Made you feel subtly threatened with some sort of retaliation for not being sexually cooperative (e.g., the mention of an upcoming evaluation, review, etc.)?
14. Touched you (e.g., laid a hand on your bare arm, put an arm around your shoulders) in a way that made you feel uncomfortable?
15. Made unwanted attempts to stroke or fondle you (e.g., stroking your leg or neck, etc.)?
16. Made unwanted attempts to have sex with you that resulted in you pleading, crying, or physically struggling?
17. Implied faster promotions or better treatment if you were sexually cooperative?
18. Made it necessary for you to respond positively to sexual or social invitations in order to be well-treated on the job?
19. Made you afraid you would be treated poorly if you didn’t cooperate sexually?
20. Treated you badly for refusing to have sex?

© 2023 Viglianti EM et al. *JAMA Network Open*.

eMethods 2. Detailed Statistical Approach

Sexual harassment experience

Self-reported sexual harassment experiences were defined as "yes" if the participant reported "once" or more frequent responses (e.g., sometimes, often, very often) to questions 2-20 from the SEQ-S survey instrument.

General approach

We used a 2-level multilevel logistic regression model to assess institutional variation in intern experiences of sexual harassment. We initially calculated the variation in the reporting of sexual harassment across institutions without adjusting for any intern characteristics ("empty model"). We subsequently created a multilevel logistic model for experiencing sexual harassment (Outcome Yes=1) adjusting for intern demographics (e.g., age, sex, race/ethnicity).

Reliability adjustments

We used reliability adjustment to avoid overestimating the probability of experiencing sexual harassment at institutions with a low number of resident responders. To do this, we used a multilevel model with a random intercept for the institution. The three main advantages of using a random effects model are (1) to reduce the number of parameters estimated, (2) to adjust for institution level covariates, and (3) to benefit from the property of shrinkage. The shrinkage estimator approach places more weight on a hospital's point estimate when it is measured reliably but "shrinks" it toward the population mean when there is more error in the measurement (e.g., lower case volume). We performed reliability adjustment by generating empirical Bayes estimates.

Quantifying the variation between academic institutions

© 2023 Viglianti EM et al. *JAMA Network Open*.

We created two multilevel logistic models. For each model, we calculated the ICC. We then directly compared the ICC for the two models (model 1 was the "empty model" and model 2 included resident demographics). The median odds ratio (MOR) was calculated from the final model as per the method of Merlo. We present MORs, as they provide more interpretable information on the odds ratio scale of the impact of hospitals on the reporting of sexual harassment by randomly comparing pairs of institutions at highest risk to those at lowest risk.

Sensitivity Analysis

To understand if there was variation by specialty training, all interns who responded to the SEQ-S questions were included and categorized into specialty training programs. The specialty training programs were categorized into 9 broad categories: internal medicine, family medicine, pediatrics, emergency medicine, general surgery and specialties (e.g., orthopedic, urology), obstetrics-gynecology, neurology, psychiatry, and other.

A similar statistical approach was used to understand if there was variation across specialties by creating a 2-level multilevel logistic regression model to assess specialty training program variation in intern experiences of sexual harassment. We initially calculated the variation in the experiences of sexual harassment across specialty programs without adjusting for any intern characteristics ("empty model"). We subsequently created a model adjusting for intern characteristics (demographics). We then directly compared the ICC for the two models (model 1 was the "empty model" and model 2 included resident demographics). The median odds ratio (MOR) was calculated from the final model as per the method of Merlo.

© 2023 Viglianti EM et al. *JAMA Network Open*.

eReference

1. Fang Y, Bohnert ASB, Pereira-Lima K, Cleary J, Frank E, Zhao Z, Dempsey W, Sen S. Trends in Depressive Symptoms and Associated Factors During Residency, 2007 to 2019: A Repeated Annual Cohort Study. *Ann Intern Med.* 2022 Jan;175(1):56-64. doi: 10.7326/M21-1594. Epub 2021 Nov 16. PMID: 34781718.

© 2023 Viglianti EM et al. *JAMA Network Open*.
