# Supplementary material for: Variation by Institution in Sexual Harassment Experiences Among US Medical Interns
Source: JAMA Netw Open. 2023 Dec 26;6(12):e2349129. doi: 10.1001/jamanetworkopen.2023.49129 (PMC10751587; doi:10.1001/jamanetworkopen.2023.49129)
Supplement: Supplement 2. — Data Sharing Statement [file jamanetwopen-e2349129-s002.pdf]

Data Sharing Statement

Viglianti. Variation by Institution in Sexual Harassment Experiences Among US Interns. *JAMA Netw Open*. Published December 26, 2023. doi:10.1001/jamanetworkopen.2023.49129

Data
Data available: No

Additional Information
Explanation for why data not available: Data available through ICPSR here: https://www.openicpsr.org/openicpsr/project/129225/version/V1/view
